# Pathways and delays in the diagnosis of autism spectrum disorder in Kenya: a cross-sectional study from tertiary hospitals in Nairobi

**DOI:** 10.1186/s13034-025-00916-2

**Published:** 2025-10-21

**Authors:** Muthoni Muthiga, Anne Mbwayo, Rachel Kang’ethe, Neil Horn

**Affiliations:** 1 Department of Psychiatry, University of Nairobi, P.O Box 30197, 00100 Nairobi, Kenya; 2 University of Cape Town, Cape Town, South Africa

**Keywords:** Autism spectrum disorder, Pathway, Delay in diagnosis

## Abstract

**Background:**

Autism Spectrum Disorder (ASD) contributes significantly to the disease burden among children and adolescents. Early diagnosis and intervention significantly improve outcomes; however, in Africa, children and adolescents with ASD are frequently identified and diagnosed late. This has been attributed to long and tortuous pathways to diagnosis. The objective of the study was to document and evaluate the pathways to a diagnosis of ASD, measure the delay in diagnosis, and document factors influencing these.

**Methods:**

A cross-sectional survey of 70 caregivers of children aged 2–18 years with ASD. The Encounter Form, developed by the World Health Organisation, was used to describe pathways to diagnosis, and structured clinical interviews and assessments were used to determine how children’s clinical factors, caregiver socio-demographic factors, and cultural and contextual factors influence the pathways and delays in diagnosis. ASD was diagnosed by a consultant psychiatrist or paediatrician using the Diagnostic and Statistical Manual version 5. SPSS version 23.0 was used for data analysis. Correlations between variables were analysed using Kruskal-Wallis, Mann-Whitney U tests, and logistic regression models.

**Results:**

A mainstream (healthcare) and traditional/spiritual-based pathway was utilized by *N* = 51 (73%) and *N* = 19 (27%) caregivers, respectively. The mean age of diagnosis was five years, with a delay of 34.9 ± 33.5 months between caregiver symptom recognition and diagnosis. A median of four points of contact was made with care providers before diagnosis, with special needs teachers serving as the primary referral source. Clinical factors associated with a delay in diagnosis included: echolalia (*p* = 0.03), delayed walking (*p* = 0.01), attention deficit hyperactivity disorder (*p* = 0.04), and intellectual developmental disorder (*p* = 0.02). Conversely, challenges in recognizing, interpreting, and responding to emotional cues (*p* = 0.03) and “selectiveness in clothing” (*p* = 0.01) were associated with an earlier diagnosis.

**Conclusion:**

Despite early recognition of ASD symptoms by caregivers and the predominant use of mainstream healthcare-based pathways, diagnosis was often delayed in this Kenyan sample. Distinct factors associated with the delay in diagnosis were identified, and further research is needed in larger and more diverse groups to facilitate earlier diagnosis and intervention.

**Supplementary Information:**

The online version contains supplementary material available at 10.1186/s13034-025-00916-2.

## Introduction

Autism Spectrum Disorder (ASD) is a complex neurodevelopmental condition characterized by restricted and repetitive behaviours, as well as challenges in social interaction and communication. ASD typically becomes evident in early childhood and ranks among the most prevalent developmental disorders affecting children [1]. The World Health Organisation (WHO) recognizes ASD as a major public health concern due to its substantial impact on the well-being of children and adolescents [2].

Although a significant proportion of ASD research has been conducted in Western countries, the majority of individuals with ASD are believed to reside in low- to middle-income countries [3]. In sub-Saharan Africa, the burden of ASD remains unknown, and a systematic review by Abubakar et al. indicates that research in this region is scant [4].

The core symptoms of ASD typically emerge between 12 and 24 months of age, and a reliable diagnosis can usually be made by the time a child is two years old [5]. Late diagnosis of ASD can lead to long-term economic, social, and emotional challenges for individuals and negatively impact caregivers’ well-being [6, 7]. However, intensive early intervention has been shown to improve language, cognition, and adaptive functioning for many children diagnosed with ASD, ultimately leading to better economic outcomes for families and communities [8, 9].

A meta-analysis and systematic review including studies from 35 countries, including low- and middle-income countries (LMICs), found that the mean age for ASD diagnosis was 60.48 months, with ages ranging from 30.90 to 234.57 months [10]. In LMICs in Africa and East Asia, ASD diagnosis is delayed compared to developed countries. This delay has been attributed to a complex and prolonged diagnostic process, often involving interactions with traditional and spiritual practitioners before seeking medical care. Limited accessibility, affordability, and acceptability of mental health services, coupled with stigma, can contribute to delays in identifying and diagnosing ASD [11–13].

Few studies have examined the age of ASD diagnosis in Africa. Research from Nigeria suggests that the mean age of ASD diagnosis is between eight and nine years [12, 14]. In Kenya, studies documenting the mean age of diagnosis vary, reporting a range from three years to nine years [15, 16].

Studies on factors affecting ASD diagnosis delay have been conducted in the United States (US), where children living in rural areas receive an ASD diagnosis 0.9 years earlier, and those with more severe communication deficits are diagnosed 1.2 years earlier [17]. In developed countries, children with comorbid intellectual and developmental disabilities (IDD) tend to receive an earlier diagnosis [17, 18]. Cultural and contextual factors influencing ASD diagnosis delay have been investigated in LMICs. In India, ASD diagnosis delay was longer in cases of comorbid epilepsy and a lack of awareness of developmental milestones among caregivers and health professionals, though prior caregiver knowledge of developmental disorders did not affect the delay [19]. In Mexico, parental concerns about language and perceived developmental delay resulted in a shorter ASD diagnosis delay [20]. A study in Nigeria did not find any clinical factors significantly associated with ASD diagnosis delay [12]. No studies on factors influencing ASD diagnosis delays have been reported in East African countries.

Understanding pathways to care is crucial for developing successful interventions that promote early detection and diagnosis of developmental disorders [21]. A cross-sectional study of 11 countries examined pathways to care in various cultural contexts. In developed countries, patients primarily sought help from a primary care medical practitioner, who referred them to mental health services. However, in LMICs, care pathways were often indirect and involved multiple providers, including traditional healers [22]. In North India, the pathway to ASD diagnosis was found to be complex and arduous, with multiple barriers and challenges, including cultural nuances, lack of awareness of ASD, and non-biological explanatory models contributing to delayed diagnosis [19].

In African countries, children and adolescents suffering from psychiatric illnesses seek care in both medical facilities and traditional/spiritual-based services. Socio-demographic variables, cultural beliefs, affordability and availability of services, and disease characteristics determine the type of pathway used and the length of time taken to receive psychiatric care [23]. Studies from Nigeria and Egypt show that complex pathways, including referrals to traditional healers, are common [23, 24]. In Kenya, traditional/spiritual-based care providers are frequently involved in pathways to care for mental health disorders [25, 26]. However, no studies in Kenya or sub-Saharan Africa have documented specific pathways to ASD diagnosis or factors influencing these pathways.

To bridge these gaps, we conducted a study describing pathways to ASD diagnosis, delays in diagnosis, and the clinical, caregiver, contextual, and cultural factors influencing them.

**Fig. 1:**
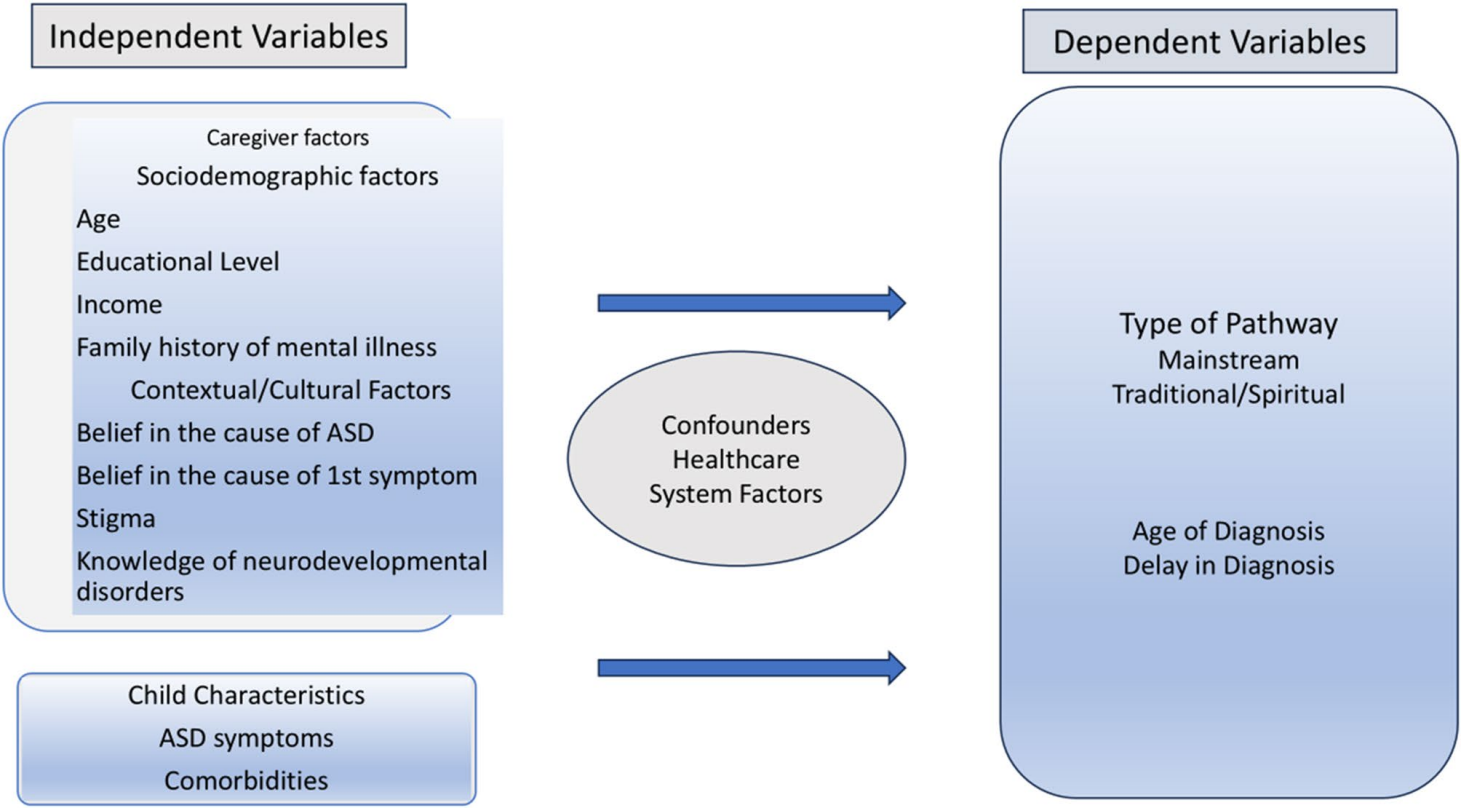
Conceptual Framework of predictors influencing pathway type and age and delay in diagnosis of ASD

The Andersen model, proposed by Walter et al., was integrated with the De Leeuw framework to provide a detailed conceptual approach for analysing ASD diagnosis delays [27, 28] (see Fig. 1). The Andersen model divides care pathways into distinct stages characterized by specific delays, accounting for various contributing factors such as clinical aspects, sociodemographic influences, and healthcare system dynamics [27]. Meanwhile, the De Leeuw framework explores cultural and contextual elements, including stigma, mental health literacy, and service accessibility, that may impact each stage [28].

## Methods

We conducted a cross-sectional survey of pathways to care and diagnosis used by caregivers of children with ASD. The study was conducted at Mathare National Teaching and Referral Hospital (MNTRH) and Kenyatta National Hospital (KNH), both in Nairobi. Caregivers of children who attended these tertiary hospitals’ Child and Adolescent Psychiatry and Occupational Therapy clinics between February and March 2023 were invited to participate in the study. Participants were eligible for the study if they were 18 years or older, cared for a child diagnosed with ASD by a paediatrician or psychiatrist with a Master’s in Medicine, and provided informed consent to participate.

We used Cochrane’s formula, adjusted for a finite population, to determine the sample size. Using a 12.7% prevalence rate from Kamau et al. [29], a 95% confidence level, and a 5% margin of error, we arrived at a sample size of 70 participants. After reviewing hospital and clinic records, we found that KNH receives twice as many ASD patients as MNTRH, so we stratified the sample in a 2:1 ratio, recruiting 47 caregivers from KNH and 23 from MNTRH. Participants were then recruited sequentially from both centres. The researcher went through the register of patients were booked to attend, and had appeared for treatment at the Child Psychiatry and Adolescent clinics and Occupational Therapy (OT) clinics in KNH and the Child Psychiatry clinic in MNTRH and using convenient sampling selected those who had a documented diagnosis of ASD made by a paediatrician or psychiatrist meeting the Diagnostic and Statistical Manual of Mental Disorders, 5th Edition (DSM– 5) criteria [30]. Of 72 caregivers approached, 70 were suitable, one did not have enough information on the sequence of care agencies the child had attended and was excluded, and one caregiver declined to participate.

The WHO Encounter Form [31] (see Additional File 2) is an instrument developed by the WHO to collect information regarding the care services patients use before and during seeking care in mental health settings. It has been used in multiple studies in LMICs, including Ethiopia, Ghana, and Bangladesh, to determine pathways to psychiatric care [32–34] and was piloted and validated in Nairobi, Kenya, in the WHO cross-cultural Pathway Study by Gater et al. in 1991 [23]. Patra et al. successfully used it to determine pathways to care for children with ASD [35]. The encounter form systematically gathers information about the initial decision to seek help, including observed symptoms, their duration, and the sources of care consulted (contacts) prior to a medical practitioner making a diagnosis. It also collects details about the professions of the individuals consulted before the diagnosis and the time elapsed from when the patient was first seen, when symptoms were first noted, to the time of diagnosis, as well as the time between each contact [31]. We tailored the encounter form to address ASD symptoms specifically and were able to record age at diagnosis, the duration from when caregivers first observed symptoms to the actual diagnosis (diagnosis delay), the interval between symptom recognition and when parents first sought assistance (parental delay), and whether traditional or spiritual healers were involved in the help-seeking process (type of pathway).

The Structured Clinical Interview and Clinical Assessment (see Additional File 1) included standard closed ended and a few open-ended questions, which included socio-demographic characteristics, family history of mental illness or developmental disorders, and the caregiver’s knowledge and beliefs concerning ASD, all variables identified in the literature as influencing age at diagnosis [20, 21, 35]. The questionnaire included a checklist of prior and observed clinical characteristics of ASD. Knowledge about developmental milestones was determined by asking the participant if they knew the age at which a child typically develops speech. If they answered ‘2 years’, then they were determined to know about developmental milestones, and the questionnaire was ticked ‘yes’. If they gave a different answer, then they were determined not to have knowledge.

Once included in the study, participants were taken through the WHO Encounter Form, the structured clinical interview, and clinical assessment. The researcher, who was a medical doctor (MBChB) and final year resident studying their Master of Psychiatry, read out the items in the questionnaire to the participants and recorded their responses verbatim. The researcher also carried out a clinical assessment and collected history on symptoms specific to ASD from direct observation. Data on clinical features that the participants could not give were collected from the physician’s notes in the patient’s files. A comorbid diagnosis, such as Intellectual Developmental Disorder and ADHD, was confirmed if a consultant psychiatrist made it and if the children met the criteria according to DSM-5.

Data was analysed using SPSS version 23. Responses to open-ended questions were analysed by identifying recurrent themes and applying thematic coding. To assess correlations with the dependent variables, themes related to the first symptom of concern and the symptom that prompted seeking help were grouped together to enhance statistical power in both bivariate and multivariate analyses. The dependent variables, age of ASD diagnosis and delay in diagnosis, were examined for normality using the Shapiro-Wilk test. Neither variable was normally distributed despite attempts at transformation. As a result, non-parametric tests were employed, specifically the Kruskal-Wallis test and the Mann-Whitney U test, to identify significant differences in associations between the independent variables and age and delay in ASD diagnosis. Mann-Whitney U Tests were conducted to identify significant differences in the age of diagnosis and the delay in diagnosis based on caregiver age, employment status, education level, knowledge of neurodevelopmental disorders, and experiences of stigma, along with child clinical characteristics. Additionally, Kruskal-Wallis Tests were utilized to assess the significant differences between age and delay in diagnosis, household income, caregiver relationship to child, county of residence, first symptom of concern, the person who saw the first symptom, belief in the cause of 1st symptom, and belief in the cause of ASD.

Logistic regression was used to examine the relationship between various predictors and the type of pathway used. We first conducted Chi-square tests to determine which predictors to include in the logistic regression analysis. Variables that demonstrated statistical significance (*p* < 0.05) were subsequently included in the binary logistic regression analysis to examine their independent predictive effects on the type of pathway. Single predictors were initially entered into the model to assess their association with the kind of pathway. Multivariate analysis was then conducted by entering all predictor variables into the equation to control for covariates. Empirical forward logistic regression was then used to determine the most reliable significant predictors of the type of pathway. The significance level was set at *p* < 0.05, and the model’s fit was confirmed through the Hosmer-Lemeshow test, which yielded a p-value of 0.91, indicating a strong fit.

This study was conducted in accordance with the principles of the Declaration of Helsinki [36]. Ethical approval was obtained from the Kenyatta National Hospital Ethics and Research Committee on February 10, 2023, under the approval number P793/10/2022. Written informed consent was obtained from all caregivers who participated in the study.

## Results

Among the 70 caregivers included in the study, 84.3% (*N* = 59) were female, and 92.9% (*N* = 65) were biological parents. Table 1 provides further details of caregiver socio-demographic characteristics and their knowledge and beliefs.

**Table 1:** Caregiver demographic, socioeconomic characteristics, knowledge and beliefs (*N* = 70)

Caregiver characteristic	Category	*N* = 70	%
Gender	Female	59	84.3
Age (years)	18–35	35	50.0
	36+	35	50.0
Relationship to child	Biological parent	65	92.9
	Parent by adoption	2	2.9
	Grandparent	3	4.3
Mother’s education level	Below secondary school	12	17.1
	Secondary school and above	58	82.9
Marital status	Married	50	71.4
	Single	20	28.6
Number of children	1–2	44	62.9
	3+	26	37.1
Employment status	Employed	27	38.6
	Unemployed	43	61.4
Household income (USD)	<=100	17	24.3
	101–500	39	55.7
	> 501	14	20.0
Source of funding for care	Self	60	85.7
	Sponsor/insurance	10	11.4
Religion	Christian	65	92.9
	Muslim	5	7.1
Person who saw 1st symptom	Family member	64	91.4
	Health worker	1	1.4
	Teacher/friends/neighbours	5	7.1
Belief in cause of 1st symptom	Biomedical	28	40.0
	Spiritual/cultural	30	42.9
	Environmental/social	10	14.3
	Don’t know	2	2.9
Believe in the cause of ASD	Don’t know	26	37.1
	Biomedical	36	51.4
	Supernatural/Cultural	8	11.4
Knowledge of developmental milestones	Yes	60	85.7
Knowledge of ASD/developmental disorder prior to diagnosis	Yes	27	38.6
Experienced stigma	Yes	51	72.9

### Child clinical descriptors and characteristics

Of the 70 children and adolescents accompanying the caregivers, 85.7% (*N* = 60) were male, with a mean age of 57.4 ± 17.2 months. Further details on ASD clinical descriptors and comorbidities are presented in Table 2.

The most frequently reported initial symptom was developmental milestone delays or regression (including speech delay), observed in 61.4% (*N* = 43) of cases, followed by impaired socio-communicative functions, such as lack of social gestures and difficulties forming relationships, in 34.3% (*N* = 24). Hyperactivity and obsessive interests were noted in 4.3% (*N* = 3). Despite these early symptoms, the primary concerns that caregivers initially sought medical intervention for, were related to aggressive behaviours, hyperactivity, or self-harming tendencies in 39% (*N* = 27) of cases, followed by speech impairment in 35% (*N* = 24), then neurodevelopmental regression and delayed milestone attainment in 21% (*N* = 15), and sociocommunicative deficits, including failure to respond when called or difficulty making friends, in 6% (*N* = 4). Speech delay was the most commonly reported developmental delay, observed in 97.1% (*N* = 64) of cases, 44.3% (*N* = 31) of caregivers reported milestone regression, while 31.4%. (*N* = 22) reported delayed walking, and 17.1% (*N* = 12) indicated unsuccessful toilet training. Caregivers first noticed developmental concerns at a mean age of 24.4 ± 9.1 months and a median of 24 months, but professional care was sought at a mean age of 38.3 ± 16.4 months and a median of 36 months, resulting in an average delay of 13.9 months before intervention. The reasons cited for delayed care-seeking included underestimating symptom severity (67.1%, *N* = 47), social and financial stressors (15.7%, *N* = 11), and preference for spiritual or cultural guidance (8.6%, *N* = 6), while 8.6% (*N* = 6) of caregivers reported no delay.

**Table 2:** Clinical characteristics of children diagnosed with ASD based on caregiver history and direct observation (*N* = 70)

Clinical Characteristic		*N* = 70	%
Gender of child	Male	60	85.7
**DSM 5 ASD Criterion A**			
Speech delay		68	97.1
Fail to respond to someone calling their name		51	72.9
Using pronouns inappropriately		7	10.0
Making little or no eye contact		61	87.1
Absence of social gestures		30	42.9
Rarely shares enjoyment for objects or activities with others		61	87.1
Has trouble understanding another person’s feelings		35	50.0
Has trouble maintaining social relationships with others		70	100.0
**DSM 5 ASD Criterion B**			
Avoids physical contact		24	34.3
Echolalia		32	45.7
Forms rows regularly		61	87.1
Abnormal movements e.g. (hand flapping, tiptoeing)		65	92.9
Has obsessive interests/routines		61	87.1
Abnormal response to pain or sound		66	94.3
Selective with clothing		33	47.1
Selective with foods		22	31.4
**Comorbidities**			
ADHD		32	45.7
IDD		18	25.7
Epilepsy		11	15.7
Learning disorder		3	4.3
Sleep problems		6	8.6
Nutritional challenges		7	10.0

### Pathways to care

**Fig. 2:**
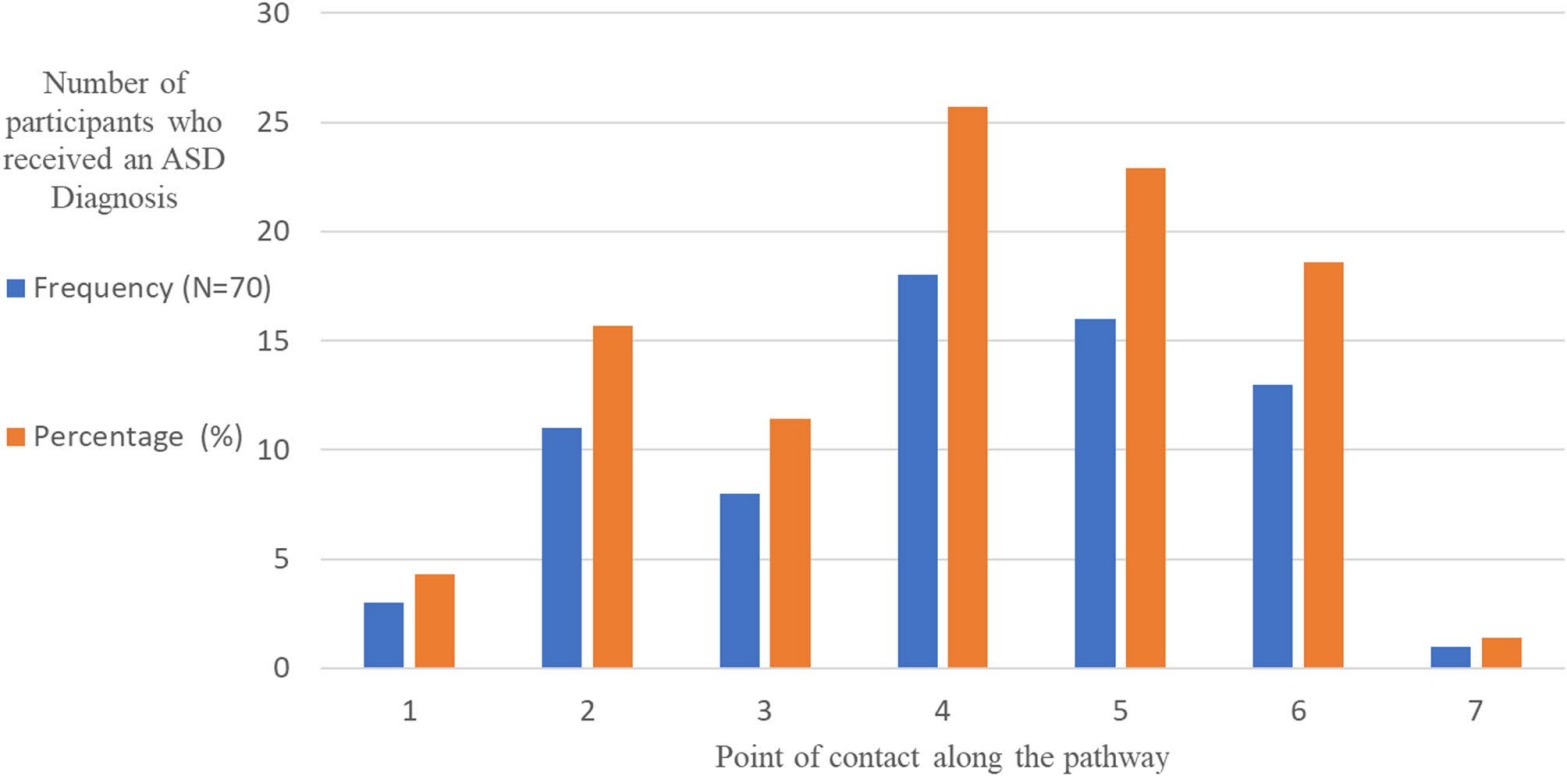
illustrates the distribution of ASD diagnoses across contact points in the pathway to care among study participants (*N* = 70). The highest number of diagnoses was received at Contact Point 4, followed by Contact Points 5 and 6

Study participants visited a median of four care providers before receiving an autism spectrum disorder (ASD) diagnosis, with the number of contact points ranging from 1 to 7 (Fig. 2). The highest number of diagnoses was received at the 4th point of contact, followed by the 5th and 6th point of contact. The majority of caregivers (71.4%, *N* = 50) reported that a psychiatrist made the diagnosis. A paediatrician diagnosed ASD in 17.1% (*N* = 12) of cases, while 10% (*N* = 7) received diagnoses from occupational or speech therapists (paraspecialists). Additionally, 1.4% (*N* = 1) of participants reported that an Ear, Nose, and Throat (ENT) surgeon made the diagnosis. Notably, none of the caregivers recalled the use of standardized diagnostic tools; however, some reported undergoing investigations such as audiometry and brain MRIs.

Among the study participants, only 8.5% (*N* = 6) received a diagnosis of ASD at their first point of contact. The majority (60%, *N* = 42) did not receive a diagnosis at this initial encounter. Instead, 12.9% (*N* = 9) were diagnosed with speech delay or delayed milestones, 12.9% (*N* = 9) were informed that their child’s symptoms were attributed to a curse or spiritual attack. 4.3% (*N* = 3) received a diagnosis of neurodevelopmental disorder while 8.6% (*N* = 6) were diagnosed with epilepsy, rickets, or speech impairment. Table 3 summarizes the cadres of care providers encountered at the first point of contact and the treatments offered.

**Table 3:** Distribution of care providers and treatment modalities for ASD at first point of care (*N* = 70)

Cadre of care providers	*N* (%)
Consultant Specialist (Psychiatrist, Paediatrician, Neurologist)	18 (25.7)
Non-specialist Health worker (general practitioner, general nurse, psychotherapist)	34 (48.6)
Para specialist (Occupational/speech therapist)	2 (2.9)
Special needs assessor (teacher in special education)	6 (8.6)
Traditional/spiritual healer	10 (14.3)
**Treatment Offered**	
Reassurance only	17 (24.3)
Referral	15 (21.4)
Medication only/surgery	2 (2.9)
Occupational/speech therapy	15 (21.4)
Nutritional counselling	2 (2.9)
Ridicule	9 (12.9)
Prayers/traditional rituals	8 (11.4)
Occupational/speech therapy and school placement recommendation	1 (1.4)
Psychotherapy	1 (1.4)

The most common intervention provided was reassurance only, reported by 24.3% (*N* = 17) of participants. Referral was offered in 21.4% (*N* = 15) of cases, while occupational and speech therapy were provided in a similar proportion.

According to the findings, two categories of pathways to care and diagnosis were identified:

1. The traditional/spiritual-based pathway involved caregivers visiting non-medical facilities, such as traditional healers and religious institutions, searching for treatment for their children.
2. The healthcare-based/mainstream pathway involved caregivers visiting ‘mainstream’ healthcare facilities but not using traditional or spiritual settings to acquire treatment for their children.

Over two-thirds of participants (72.9%, *N* = 51) used the healthcare-based pathway, while 27.1% (*N* = 19) adopted the traditional/spiritual pathway.

### Factors associated with pathway selected by caregivers

Supplementary Tables 1, 2, and 6 in Additional File 3 present the bivariate analysis results of factors associated with pathway selection. Caregivers who reported experiencing stigma or attributed the first symptom they noticed in their child to spiritual or cultural causes were significantly more likely to choose the traditional/spiritual pathway. Conversely, caregivers with prior knowledge of neurodevelopmental disorders or those who believed ASD was attributed to biomedical factors were more inclined to opt for the mainstream healthcare pathway. Stigma was strongly associated with pathway selection (OR = 9.82), while prior knowledge of neurodevelopmental disorders was linked to an OR of 8.17. Beliefs regarding the cause of the first symptom and ASD both had an OR of 4.9.

Socio-demographic characteristics, including caregiver age, maternal educational level, family history of mental illness, income, and number of children per household, did not significantly predict pathway selection, nor did the first symptom of concern or the symptom that prompted the caregivers to seek help. However, children with comorbid ADHD and IDD were significantly more likely to follow the traditional/spiritual pathway (see Table 4, found at the end of the document).

**Table 4:** Univariate logistic regression analysis of factors associated with the type of pathway

		Traditional/spiritual pathway		Mainstream pathway	
	p-value	OR	95% CI	OR	95% CI
**Caregiver characteristics**					
Older age (36+)	0.79	0.87	0.30–2.48	1.16	0.40–3.32
Maternal educational level (secondary and above)	0.06	0.29	0.08–1.05	3.47	0.95–12.57
Employment status (employed)	0.46	1.52	0.50–4.63	0.66	0.22–2.01
Higher household income (>$501)	0.14	0.42	0.13–1.33	2.32	0.75–7.63
Stigma	***0.03***	***9.82*** ^a^	***1.21–79.7***	*0.1*	*0.01–0.83*
Belief in the cause of 1st symptom (spiritual/cultural)	***0.04***	***4.90*** ^a^	***1.1–22.2***	*0.20*	*0.05–0.94*
Knowledge of Neurodevelopmental Disorders	*0.01*	*0.12*	*0.03–0.58*	***8.17*** ^b^	***1.71–39.07***
Belief in the cause of ASD (Biomedical)	*0.03*	*0.21*	*0.05–0.90*	***4.90*** ^b^	***1.15–20.70***
**Child characteristics**					
ADHD	***0.02***	***3.65*** ^a^	***1.19–11.20***	*0.27*	*0.09–0.84*
IDD	***0.01***	***4.2*** ^a^	***1.33–13.3***	*0.24*	*0.08–0.75*
Epilepsy	0.46	1.68	0.43–6.54	0.59	0.15–2.33

The variables presented in Table 4 were selected based on existing literature demonstrating their influence on help-seeking behaviour. Additionally, only variables where chi-square tests yielded p-values < 0.05 were included (see additional file 3). Increased odds of using the traditional/spiritual pathway, ^b^Increased odds of using the mainstream healthcare pathway

After adjusting for covariates, stigma remained a significant predictor of traditional/spiritual pathway use (AOR = 8.57, *p* = 0.049, 95% CI: 1.01–72.71), while prior knowledge of neurodevelopmental disorders remained significantly associated with selecting the mainstream pathway (AOR = 8.17, *p* = 0.01, 95% CI: 1.71–39.07).

### Factors influencing age at ASD diagnosis and diagnostic delay

The mean age at ASD diagnosis was 59.5 ± 34.2 months, with a median of 48.5 months. The average diagnostic delay, i.e., the time between caregivers recognizing the first symptom and obtaining a formal diagnosis, was 34.9 ± 33.5 months, with a median of 26.5 months. The nature of the initial symptom did not significantly affect the timing or delay of diagnosis, nor did the symptom that prompted the caregivers to seek help (see supplementary Table 5 in additional file 3). Children from higher-income households and those whose caregivers had prior knowledge of neurodevelopmental disorders were diagnosed earlier and experienced shorter diagnostic delays. In contrast, children whose caregivers attributed ASD to supernatural causes had a later diagnosis. Similarly, children of younger caregivers received earlier diagnoses. Children with echolalia, delayed walking, and comorbid with IDD and ADHD experienced delayed diagnoses. In contrast, those whose caregivers reported difficulties in understanding others’ emotions or noted selectivity in clothing preferences received earlier diagnoses.

Table 5 presents caregiver and child characteristics significantly associated with age at diagnosis and diagnostic delay, while supplementary Tables 3 and 4 in Additional File 3 provide correlation analyses and distribution patterns for caregiver and child characteristics, respectively.

**Table 5:** Caregiver and clinical factors associated with age at ASD diagnosis and diagnostic delay (*N* = 70)

Caregiver characteristic		Age at diagnosis (Months)			Diagnostic delay (Months)		
		Median	Range	*P* value	Median	Range	*P* value
Age (years)	18–35	46.0	72.0	***0.04***	25.00	56.00	0.25
	36+	60.0	201.0		34.00	213.00	
Household income (USD)	<=100	60.0	168.0	***0.03***	37.00	166.00	***0.01***
	101–500	48.0	204.0		26.00	212.00	
	> 501	39.0	69.0		15.50	45.00	
Have you heard of ASD before diagnosis	No	60.0	201.0	***0.04***	36.00	213.00	***0.02***
	Yes	40.0	72.0		23.00	68.00	
What do you believe is the cause of ASD	Don’t know	49.5	173.0	***0.01***	28.50	172.00	***0.01***
	Biomedical	45.0	72.0		24.00	57.00	
	Supernatural	72.0	180.0		53.00	192.00	
**Clinical Characteristics**							
Has trouble understanding others’ feelings	No	60	180	***0.03***	34	172	0.10
	Yes	44	201		24	213	
Echolalia	No	45	204	0.02	24	213	0.03
	Yes	60	177		36	174	
Delay in walking	No	48	72	0.07	24	69	***0.01***
	Yes	60	197		36.5	207	
Selective with clothing	No	45	84	***0.01***	24	69	***0.02***
	Yes	60	194		37	212	
Comorbid ADHD	No	43	204	***0.04***	23	212	0.05
	Yes	56	81		36	69	
Comorbid IDD	No	47	180	***0.02***	24	175	***0.01***
	Yes	72	192		56	204	

## Discussion

This cross-sectional survey revealed that, despite caregivers predominantly using mainstream healthcare pathways and early recognition of ASD symptoms, the diagnosis of ASD in children was delayed by 3 years. Socioeconomic and cultural factors, alongside specific clinical characteristics, contributed to these delays and influenced whether caregivers sought help from traditional and spiritual healers.

Prior caregiver knowledge of ASD was associated with an earlier diagnosis, suggesting that equipping caregivers with education on ASD and implementing targeted policies and strategies to improve caregiver awareness could be instrumental in facilitating timely diagnosis. Notably, this finding differs from research conducted in Nigeria and India, where previous awareness did not result in earlier detection. In those situations, caregivers’ interpretations of their child’s symptoms frequently did not align with their comprehension of autism, which may have impacted their willingness to seek help [12, 19], while in this study caregivers frequently believed that ASD was attributed to biomedical causes and hence use of the mainstream pathway thus contributing to earlier diagnosis.

The mean age of ASD diagnosis in this study is 59.5 (± 34.2) months, similar to the global mean age of 60.48 months [10]. In Nigeria, the mean diagnostic ages vary from 9.45 ± 4.33 years [13] to 44.7 months, with a mean delay of 22.2 months [39]. Similar variations were observed in Kenyan studies. Samia et al. documented a median diagnostic age of three years in a hospital serving individuals from a higher socioeconomic background [16], whereas in a qualitative study, Kamau et al. found a later mean diagnostic age of 9–10 years [30]. Our findings underscore the significant influence of socioeconomic status on ASD diagnosis timing, with children from higher-income households receiving earlier diagnoses due to improved access to specialized care, aligning with trends observed in high-income countries [38]. This highlights a critical gap in Kenya’s newly implemented Social Health Authority (SHA), which facilitates universal health coverage but does not explicitly include ASD or neurodevelopmental disorder-related services [39]. Expanding SHA’s coverage to encompass ASD-specific interventions could be instrumental in reducing diagnostic delays and ensuring early intervention, ultimately improving long-term outcomes for affected children.

To the best of our knowledge, this is the first study to demonstrate that children exhibiting echolalia and delayed walking tend to receive a later diagnosis, whereas those who struggle to understand others’ emotions and display high selectivity in clothing choices are diagnosed earlier. The delayed diagnosis in children with echolalia may be explained by caregivers interpreting repetitive speech patterns as part of typical language development, thereby postponing recognition of atypical behaviours. In contrast, children who are highly selective with clothing may experience heightened sensitivity to pain, and this was attributed to physicians focusing on organic causes of pain rather than disorders of neurodevelopment [17]. The unexpected association between clothing preferences, poor social-emotional reciprocity, and earlier diagnosis in our study warrants further investigation. Potential cultural influences, diagnostic biases among healthcare professionals, and caregiver perceptions in identifying and reporting symptoms may play a role. Additionally, our small sample size could have influenced these findings, highlighting the need for further research to validate and explore these patterns.

Unexpectedly, children exhibiting attention and hyperactivity issues, intellectual impairments, and delays in walking received their diagnoses significantly later, which is opposite to patterns noted in other studies from high-income nations [17, 18]. This delay and that caused by delayed walking could be explained by the overlapping symptomatology of these comorbidities, which may obscure ASD-specific signs. Additionally, limited ASD knowledge among health professionals in Kenya may lead to missed early symptoms, delaying diagnosis.

Our findings indicate that the primary diagnostic pathway for ASD in Kenya relies on mainstream medical systems, which may be attributed to the urban setting of the study. However, consistent with other African studies, a notable proportion of caregivers also sought help from traditional and spiritual healers. Kamau et al. previously identified stigma as a significant barrier to appropriate autism diagnosis, treatment, and management in Kenya [17]. This study confirms that finding, showing that caregivers who reported experiencing stigma were significantly more likely to use traditional and spiritual pathways. Prior research conducted at the Kenyan Coast has documented cultural beliefs that associate ASD and other neurodevelopmental disorders with witchcraft, evil spirits, and curses [25], which likely explains the link between stigma and help-seeking in non-medical settings observed in this study. On the other hand, caregivers who had prior knowledge of neurodevelopmental disorders mainly used healthcare routes. This underscores a critical necessity for enhancing psychoeducation and reducing stigma, as these initiatives could motivate caregivers to pursue early diagnoses and suitable treatment for children with ASD.

In this study, caregivers of children with ADHD and IDD were more likely to seek care from traditional and spiritual healers, indicating that cultural perceptions of the behaviours specific to these conditions may influence their choice of care pathways. Our results are in keeping with previous research that indicated that intellectual disabilities are associated with help-seeking at traditional agencies [37]. The externalizing symptoms of ADHD—such as impulsivity and aggression—often contribute to increased family distress, potentially leading caregivers to seek non-medical interventions for solace. Epilepsy, however, was not linked to caregivers seeking traditional or spiritual healers in this study, a finding that contradicts trends observed in LMICs such as Nigeria and India [35, 37]. One potential explanation for this discrepancy is that epilepsy, like ASD, may be more widely recognized as a biomedical condition in Kenya compared to ADHD and IDD. These findings, alongside insights into caregiver perceptions of neurodevelopmental disorders, highlight how beliefs influence care-seeking behavior and alternative treatment pathways. This underscores the need for targeted psychoeducation and public awareness campaigns. Collaborations with traditional and spiritual healers could be instrumental in shifting caregiver reliance toward mainstream healthcare services, facilitating earlier diagnosis and intervention.

### Limitations

This study has several limitations that should be acknowledged. First, the relatively small sample size may reduce the ability to accurately reflect true associations, increase statistical variability, and limit the generalizability of findings to broader populations. As smaller samples may yield less precise estimates and potentially obscure subtle effects or associations present in larger datasets, future research with larger sample sizes is recommended to confirm and expand upon these findings.

Second, the study was conducted in specialized hospital settings within urban areas, which may not be representative of the general population, particularly those in rural regions with limited access to tertiary healthcare services. Thus, the applicability of findings to broader populations should be considered with caution.

Third, limitations in measures used may have affected the accuracy of diagnostic findings. We did not have access to specialized instruments for evaluating children’s IQ and ASD severity, despite evidence that these factors significantly impact diagnostic timelines. Additionally, the structured clinical interview and assessment were not piloted or formally validated. Similarly, more specific and sensitive diagnostic tools, such as the Autism Diagnostic Observation Schedule (ADOS), which is considered the gold standard for ASD diagnosis, were not utilized. Future studies should incorporate ADOS to provide a more comprehensive assessment of clinical symptoms.

Fourth, the cross-sectional design of this study has its own limitations. A longitudinal study would be beneficial for understanding how ASD diagnostic trajectories change and identifying factors that may influence pathway decisions over extended periods.

Recall bias may also have affected findings, as the WHO Encounter Form requires participants to report detailed information about events in the distant past. Additionally, social desirability bias may have influenced responses, particularly when caregivers were asked about cultural beliefs and practices related to ASD diagnosis. Furthermore, caregiver knowledge of developmental milestones may have been influenced by interactions with healthcare professionals, potentially affecting the accuracy of reported experiences. Help-seeking behaviour is inherently complex and multifaceted, shaped by numerous interrelated factors beyond the scope of this study. The lack of a defined conceptual framework that targets explicitly health-seeking behaviours reduces the reliability of these findings.

Despite these limitations, this study provides valuable insights into ASD diagnostic pathways and highlights critical areas for future research and policy interventions aimed at improving early ASD identification and care.

### Implications and future directions

This study identifies key factors contributing to significant gaps in the diagnosis of ASD, caregiver understanding, and access to specialized care. It highlights the urgent need for policy reforms within Kenya’s Mental Health Policy [39, 40], which currently lacks specific strategies for autism and other neurodevelopmental disorders. Our findings highlight the urgent need for Kenya’s Mental Health Policy to incorporate specific strategies for ASD and neurodevelopmental disorders. Key interventions could include early screening in primary care, culturally appropriate awareness programs to reduce stigma and enhance public understanding, and expanded training for healthcare workers in autism diagnosis and management. Strengthening these policy frameworks would facilitate timely identification and improve long-term outcomes for children with ASD.

## Electronic supplementary material

Below is the link to the electronic supplementary material.

Supplementary Material 1: Structured Clinical Interview and Assessment.

Supplementary Material 2: WHO Encounter Form.

Supplementary Material 3:Caregiver and clinical characteristics associated with the type of pathway, age, and delay of ASD diagnosis.

## Data Availability

The datasets used and/or analysed during the current study are available from the corresponding author MM upon reasonable request. The data are not publicly available due to restrictions, as they contain information that could compromise the privacy of research participants.

## Abbreviations

ADHD: Attention deficit hyperactivity disorder
ASD: Autism spectrum disorder
CI: Confidence interval
DSM: 5–Diagnostic and statistical manual of mental disorders, 5th edition
IDD: Intellectual developmental disorder
IQ: Intelligence quotient
KNH: Kenyatta national hospital
KSH: Kenyan shilling
LMICs: Low to middle income countries
MNTRH: Mathare national teaching and referral hospital
OR: Odds ratio
SPSS: Statistical package for the social sciences
UK: United Kingdom
US: United States
USD: United States Dollar
WHO: World Health Organisation
