# Supplementary material for: Pathways and delays in the diagnosis of autism spectrum disorder in Kenya: a cross-sectional study from tertiary hospitals in Nairobi
Source: Child Adolesc Psychiatry Ment Health. 2025 Oct 21;19:114. doi: 10.1186/s13034-025-00916-2 (PMC12539052; doi:10.1186/s13034-025-00916-2)
Supplement: Supplementary file 1 — Supplementary Material 1: Structured Clinical Interview and Assessment. [file 13034_2025_916_MOESM1_ESM.doc]

STUDYPARTICIPANTINDENTIFICATIONNUMBER……………………………….

**PART 1: SOCIODEMOGRAPHIC CHARACTERISTICS**:

1. Age in years……………………

2. Sex Male Female (tick where appropriate)

3. Relationship to the patient (tick where appropriate)

Biological Parent

Parentbyadoption

Grandparent

Other (specify)…………………………………………………………………….

3. Marital status

Single

Married

Separated

Divorced

Widowed

Cohabiting

4. Highest level of education of mother.

No formal education

Primary

Secondary

Tertiary (college and university)

5. Occupation.

Student

Formalemployment

Business person

Unemployed

Others

Specify

6. Income per month (in KSH)

<5,000

5,000-10,000

10,000-20,000

20,000-50,000

>50,000

8. Source of funding for care

Self

Sponsor

Insurance

7.Religion.

Christians

Muslims

Others

Specify………………………………

8. Area of residence (according to the older Kenyan’s provinces).

Nairobi

Central

Riftvalley

Eastern

Western

Coast

Nyanza

North Eastern.

10. Family history of mental illness/developmental disorder.

YES

NO

IF YES, which mental/developmental disorder………………………………………………………………

**PART 2: Decision to first seek help and diagnosis.**

At what age was your child/adolescent diagnosed with ASD?.........................................

How long ago was the diagnosis made?.......................................................................

Who made the diagnosis?............................................................................................

At which facility was the diagnosis made?...............................................................

Who first showed concern about the child’s development/symptoms

……………………………………………………………………………………………………

What was the first symptom or sign of concern?

…………………………………………………………………………………………………

How long ago? (In months) ….………………………………………

At what age did you notice the first symptom/sign of concern?

………………………………………………………………………………………………………

What did you believe was the cause of the first symptoms? (Choose the single best response)

Biomedical…….

Spiritual/cultural……….

Don’t know………………………..

At what age did you decide to first seek help regarding the symptoms/signs of concern? …………………………………………………………………………………………………

If you did not seek help immediately why? ………………………………………………………………………………………………

PART 3: Caregiver beliefs and knowledge of ASD

Have you heard of ASD before?

Yes No

What do you believe is the cause of ASD? (Choose single best answer)

Biomedical/environmental

Supernatural

Don’t know

‘’A child should be able to combine two words into simple phrases by the age of 2?"

Yes No

Have you heard of ASD or any other developmental disorder before your child was diagnosed with ASD?

Yes No

If yes, where?............................................................................................................................................

Has stigma affected how you have sought care for your child with ASD?

Yes No

If yes, how?...............................................................................................................................

Clinical Assessment of child with ASD

Autism Spectrum Disorder DSM-5 Diagnostic Criteria

Criterion A: Persistent deficits in social communication and social interaction across multiple contexts, as manifested by the following

A1: Deficits in social-emotional reciprocity

Language delay

Fail to respond to someone calling their name

Uses pronouns inappropriately

A2: Deficits in nonverbal communicative behaviours used for social interaction

Make little or no eye contact

Absence of social gestures e.g, waving goodbye

A3: Deficits in developing, maintaining, and understanding relationships

Rarely shares enjoyment for objects or activities with others

Has trouble understanding another person’s feelings

Have trouble maintaining social relationships with their peers

Criterion B: Restricted, repetitive patterns of behavior, interests, or activities:

B1: Stereotyped or repetitive motor movements, use of objects, or speech

Avoid physical contact

Forms rows regularly

Abnormal body movements (e.g., hand flapping, tiptoeing)

B2: Insistence on sameness, inflexible adherence to routines, or ritualized patterns of

verbal or nonverbal behaviour

Has obsessive interests

Repeat words that have been spoken

B3: Highly restricted, fixated interests that are abnormal in intensity or focus

Have obsessive routines

B4: Hyper- or hyporeactivity to sensory input or unusual interest in sensory aspects of

the environment

Have an abnormal response to sound or pain?

Selective about his/her clothes

Selective about his/her food

Clinical comorbidities

Neurodevelopmental regression

ADHD

IDD

Delay in walking

Encopresis/Enuresis

Sleep problems

Epilepsy
