# Supplementary figures and images for: Pathways and delays in the diagnosis of autism spectrum disorder in Kenya: a cross-sectional study from tertiary hospitals in Nairobi
Source: Child Adolesc Psychiatry Ment Health. 2025 Oct 21;19:114. doi: 10.1186/s13034-025-00916-2 (PMC12539052; doi:10.1186/s13034-025-00916-2)

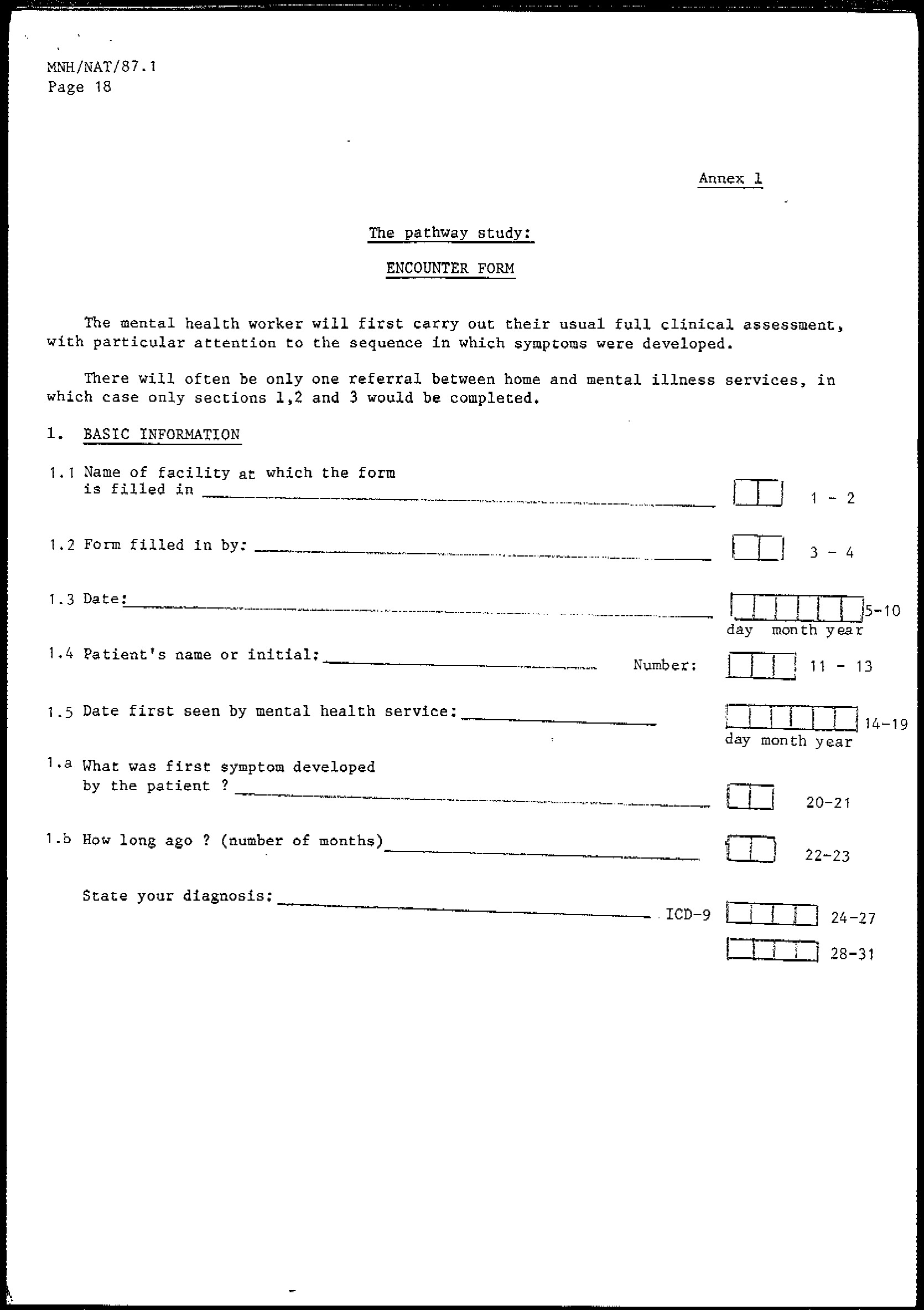


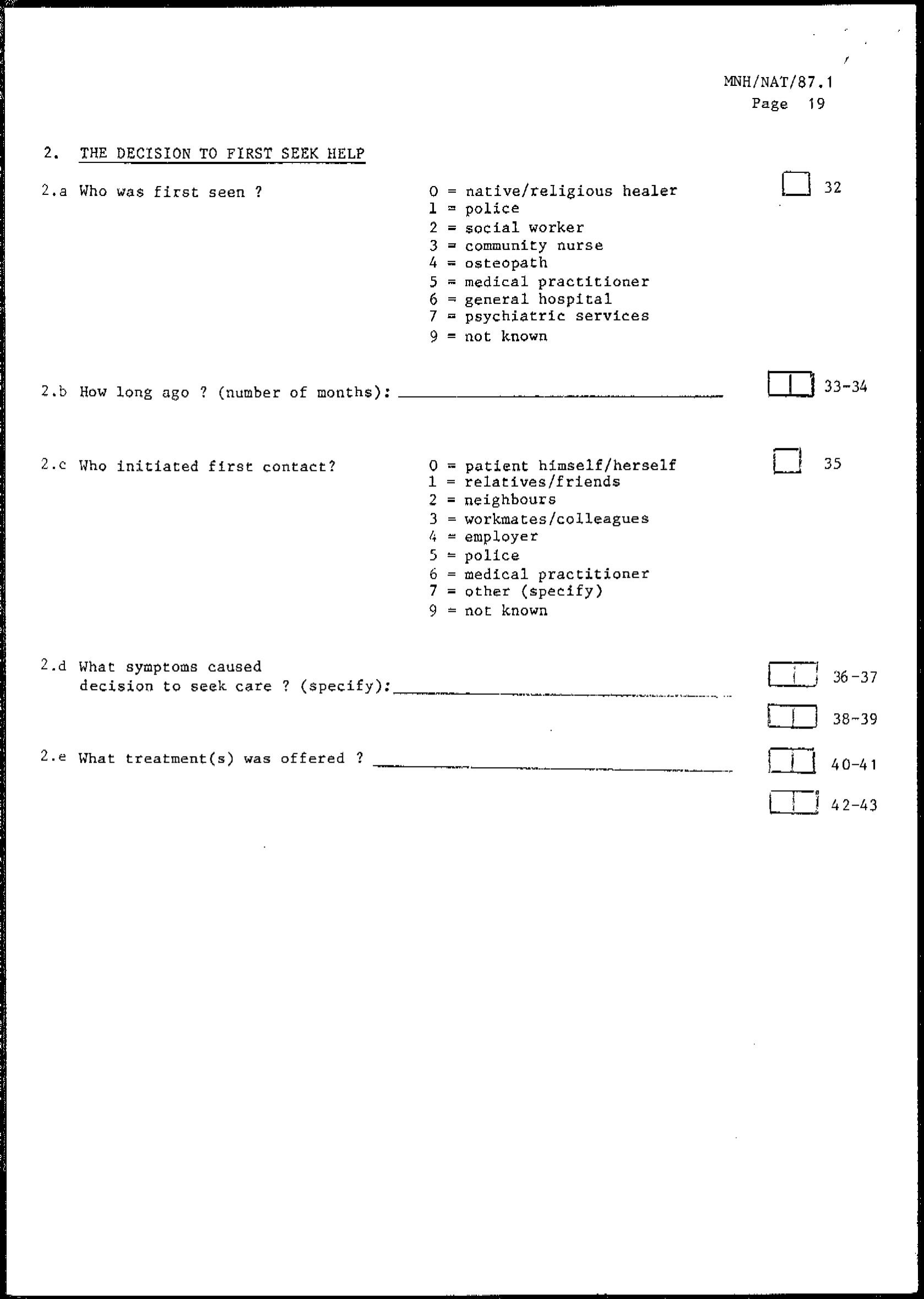


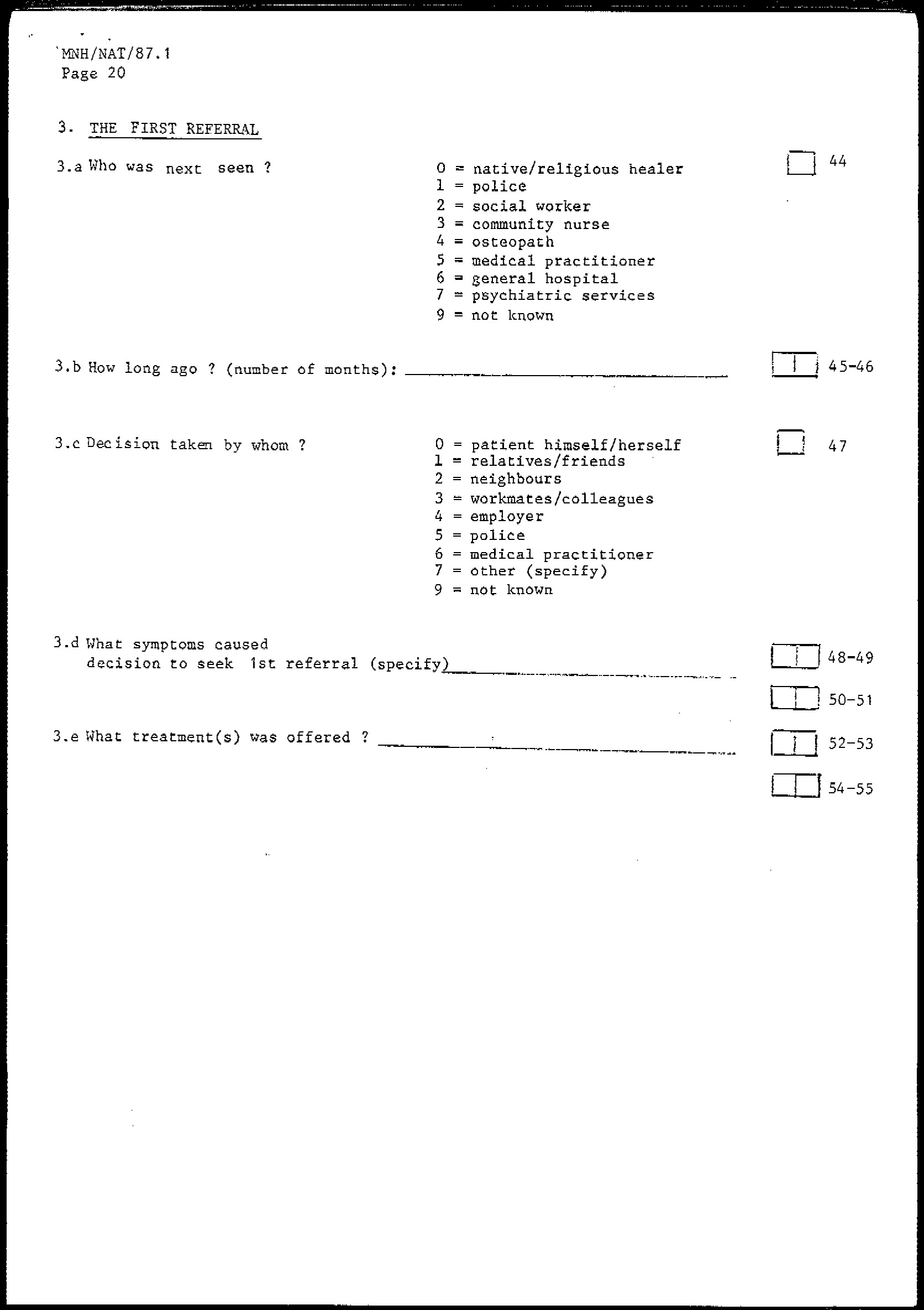


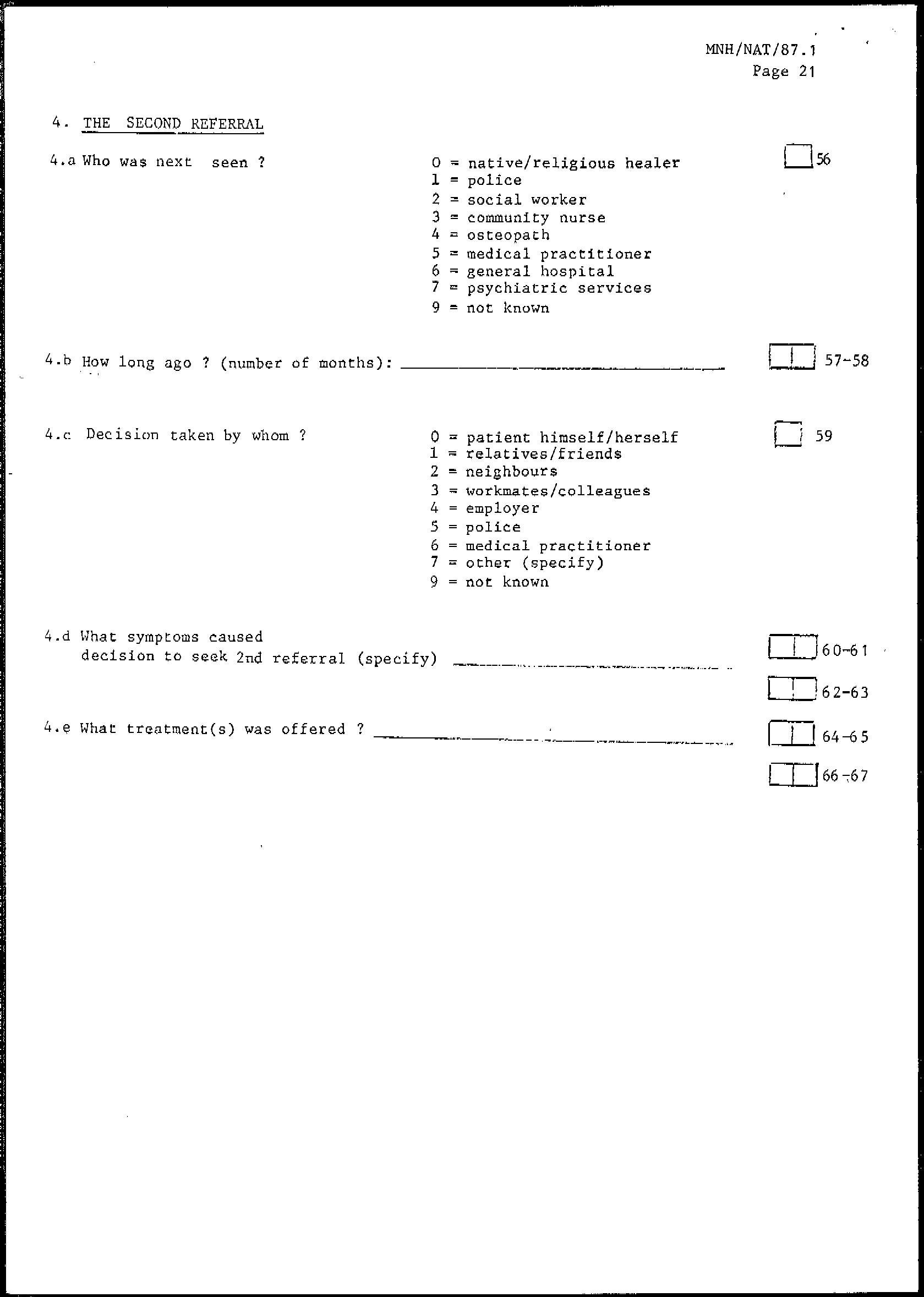


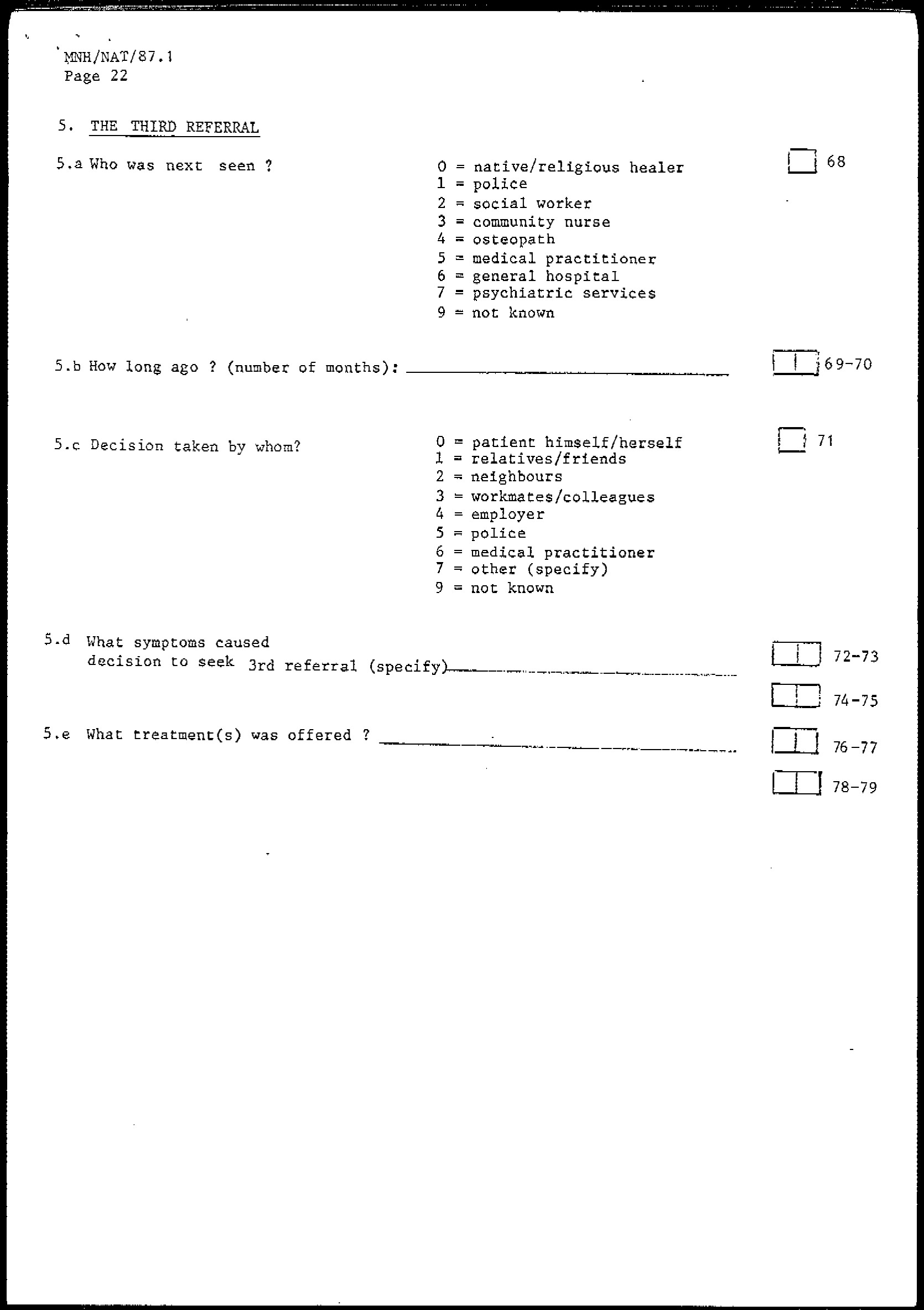


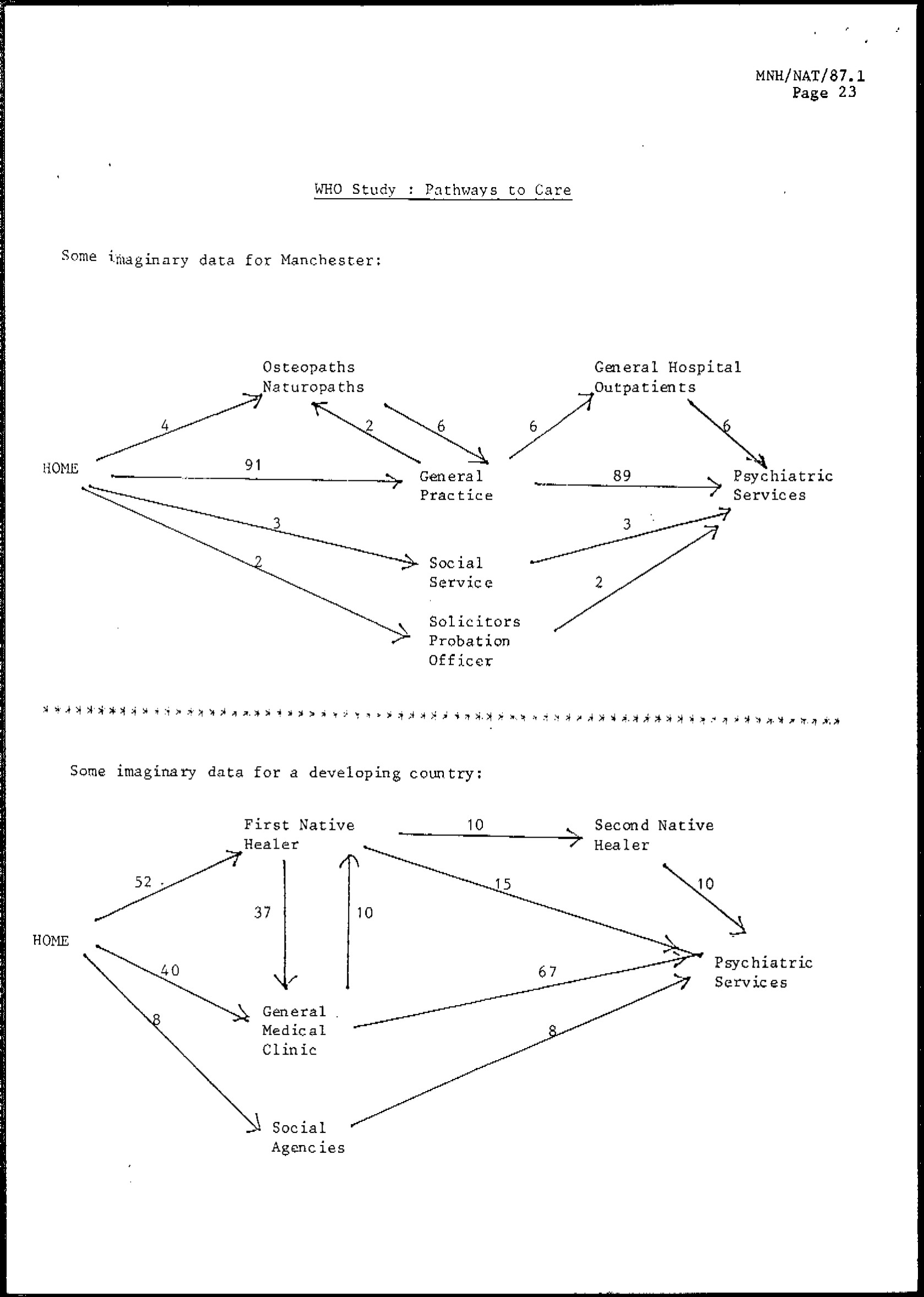

Supplement: Supplementary file 2 — Supplementary Material 2: WHO Encounter Form. [file 13034_2025_916_MOESM2_ESM.docx]
