# Supplementary material for: Pathways and delays in the diagnosis of autism spectrum disorder in Kenya: a cross-sectional study from tertiary hospitals in Nairobi
Source: Child Adolesc Psychiatry Ment Health. 2025 Oct 21;19:114. doi: 10.1186/s13034-025-00916-2 (PMC12539052; doi:10.1186/s13034-025-00916-2)
Supplement: Supplementary file 3 — Supplementary Material 3:Caregiver and clinical characteristics associated with the type of pathway, age, and delay of ASD diagnosis. [file 13034_2025_916_MOESM3_ESM.doc]

**Supplementary Table 1: Association Between Type of Pathway and Caregiver Characteristics (N=70)**

| Caregiver characteristic | Traditional/spiritual  (N=19, 27.1%) | Mainstream  (N=51, 72.9%) | Chi-square  (p-value) |
| --- | --- | --- | --- |
| **Gender** |  |  | 0.72 |
| Male | 2 | 9 |  |
| Female | 17 | 42 |  |
| **Caregiver age** |  |  | 0.79 |
| 18-35 | 10 | 25 |  |
| 36+ | 9 | 26 |  |
| **Mother’s education level** |  |  | **0.05** |
| Below secondary school | 6 | 6 |  |
| secondary school and above | 13 | 45 |  |
| **Marital status** |  |  | 0.77 |
| Married/cohabiting | 13 | 37 |  |
| Single | 6 | 14 |  |
| **Number of children** |  |  | 0.55 |
| 1 - 2 | 9 | 35 |  |
| 3+ | 10 | 16 |  |
| **Household income (USD)** |  |  | 0.09 |
| </=100 | 7 | 10 |  |
| 101-500 | 10 | 29 |  |
| >501 | 2 | 12 |  |
| **Source of funding for care** |  |  | 0.11 |
| Self | 19 | 41 |  |
| Sponsor/insurance | 0 | 10 |  |
| **Religion** |  |  | 0.31 |
| Christian | 19 | 46 |  |
| Muslim | 0 | 5 |  |
| **County of Residence** |  |  | 0.20 |
| Nairobi | 7 | 26 |  |
| Kiambu | 7 | 13 |  |
| Machakos | 4 | 3 |  |
| Nyeri | 0 | 0 |  |
| Kakamega | 0 | 0 |  |
| Muranga | 1 | 6 |  |
| Kajiado | 0 | 3 |  |
| **Family history of mental illness/neurodevelopmental disorder** |  |  | 0.57 |
| Yes | 7 | 15 |  |
| No | 12 | 36 |  |
| **Person who identified 1^st^ symptom** |  |  | 0.25 |
| Family member | 16 | 48 |  |
| Health worker | 0 | 1 |  |
| Teacher/friends/neighbours | 3 | 2 |  |
| **First symptom of concern** |  |  | 0.70 |
| Speech delay | 7 | 17 |  |
| Delayed/neurodevelopmental regression | 6 | 12 |  |
| Impaired sociocommunicative interaction, poor eye contact, difficulty making friends, etc. | 5 | 20 |  |
| Hyperactivity/obsessive interests | 1 | 2 |  |
| **Belief about the cause of the first symptom** |  |  | **<0.001** |
| Biomedical | 3 | 25 | **<0.001** |
| Spiritual/cultural | 16 | 14 |  |
| Don’t know | 0 | 12 |  |
| **Knowledge of ASD before diagnosis** |  |  | **0.005** |
| No | 17 | 26 |  |
| Yes | 2 | 25 |  |
| **Belief about the cause of ASD** |  |  | **<0.001** |
| Don't know | 8 | 18 |  |
| Biomedical | 3 | 33 |  |
| Supernatural | 8 | 0 |  |
| **Knowledge of developmental milestones** |  |  | 0.44 |
| No | 4 | 6 |  |
| Yes | 15 | 45 |  |
| **Experienced stigma** |  |  | **0.01** |
| No | 1 | 18 |  |
| yes | 18 | 33 |  |

**Supplementary Table 2: Association Between Child Characteristics and Type of Pathway Among Children (N=70)**

|  | | Type of Pathway (N=70) | |  |
| --- | --- | --- | --- | --- |
|  |  | Traditional/spiritual route | Mainstream | Chi-square test  P value |
| Gender of child | Male | 17 | 43 | 0.72 |
|  | Female | 2 | 8 |  |
| Makes little or no eye contact | No | 2 | 7 | 0.54 |
|  | Yes | 17 | 44 |  |
| Fail to respond to their name being called | No | 4 | 15 | 0.56 |
|  | Yes | 15 | 36 |  |
| Rarely shares enjoyment for objects/activities with others | No | 1 | 8 | 0.45 |
|  | Yes | 18 | 43 |  |
| Has trouble understanding others' feelings | No | 9 | 26 | 0.50 |
|  | Yes | 10 | 25 |  |
| Uses pronouns inappropriately | No | 16 | 47 | 0.40 |
|  | Yes | 3 | 4 |  |
| Echolalia | No | 9 | 29 | 0.59 |
|  | Yes | 10 | 22 |  |
| Has trouble maintaining social relationships with peers | No | 0 | 0 |  |
|  | Yes | 19 | 51 |  |
| Shows absence of social gestures | No | 10 | 30 | 0.78 |
|  | Yes | 9 | 21 |  |
| Avoids physical contact | No | 11 | 35 | 0.41 |
|  | Yes | 8 | 16 |  |
| Has obsessive interests | No | 3 | 6 | 0.69 |
|  | Yes | 16 | 45 |  |
| Abnormal response to pain and/or sound | No | 0 | 4 | 0.57 |
|  | Yes | 19 | 47 |  |
| Forms rows regularly | No | 2 | 7 | 0.72 |
|  | Yes | 17 | 44 |  |
| Successful potty training | No | 15 | 39 | 0.83 |
|  | Yes | 4 | 12 |  |
| Selective his/her clothes | No | 9 | 28 | 0.60 |
|  | Yes | 10 | 23 |  |
| Has abnormal movements | No | 0 | 5 | 0.31 |
|  | Yes | 19 | 46 |  |
| Delay in speaking | No | 0 | 2 | 0.53 |
|  | Yes | 19 | 49 |  |
| Delay in walking | No | 15 | 33 | 0.39 |
|  | Yes | 4 | 18 |  |
| Comorbid ADHD | No | 6 | 32 | ***0.03*** |
|  | Yes | 13 | 19 |  |
| Comorbid convulsive disorder | No | 15 | 44 | 0.47 |
|  | Yes | 4 | 7 |  |
| Comorbid sleep problems | Yo | 17 | 47 | 0.66 |
|  | yes | 2 | 4 |  |
| Enuresis/Encopresis | No | 15 | 43 | 0.72 |
|  | Yes | 4 | 8 |  |
| Comorbid Intellectual developmental disorder | No | 10 | 42 | ***0.03*** |
|  | Yes | 9 | 9 |  |
| Nutritional Challenges | No | 16 | 47 | 0.38 |
|  | yes | 3 | 4 |  |
| Comorbid Learning disabilities | No | 17 | 50 | 0.18 |
|  | Yes | 2 | 1 |  |

**Supplementary Table 3: Caregiver factors associated with age and delay of ASD diagnosis**

|  | | Age at diagnosis | | | Diagnostic delay time | | |
| --- | --- | --- | --- | --- | --- | --- | --- |
|  |  | Median | Range | P value | Median | Range | P value |
| Age | 18-35 | 46.0 | 72.0 | ***0.04*** | 25.00 | 56.00 | 0.25 |
|  | 36+ | 60.0 | 201.0 |  | 34.00 | 213.00 |  |
| Household income (USD) | <=100 | 60.0 | 168.0 | ***0.03*** | 37.00 | 166.00 | ***0.01*** |
|  | 101-500 | 48.0 | 204.0 |  | 26.00 | 212.00 |  |
|  | >501 | 39.0 | 69.0 |  | 15.50 | 45.00 |  |
| Religion | Christian | 48.0 | 204.0 | 0.37 | 26.00 | 213.00 | 0.38 |
|  | Muslim | 78.0 | 174.0 |  | 48.00 | 170.00 |  |
| County of residence | Nairobi | 50.0 | 177.0 | 0.34 | 34.00 | 175.00 | 0.21 |
|  | Kiambu | 48.0 | 204.0 |  | 24.00 | 212.00 |  |
|  | Machakos | 60.0 | 56.0 |  | 36.00 | 48.00 |  |
|  | Muranga | 48.0 | 32.0 |  | 24.00 | 32.00 |  |
|  | Kajiado | 36.0 | 12.0 |  | 19.00 | 18.00 |  |
| Employment status | Unemployed | 43 | 84 | 0.07 | 25 | 68 | 0.37 |
|  | Employed | 54 | 201 |  | 30 | 213 |  |
| Number of children | 1 - 2 | 48 | 200 | 0.39 | 25.5 | 212 | 0.78 |
|  | 3+ | 56 | 180 |  | 31 | 175 |  |
| Family history of mental illness/neurodevelopmental disorder | Yes | 57.0 | 198.0 | 0.62 | 36.00 | 210.00 | 0.67 |
|  | No | 48.0 | 180.0 |  | 25.00 | 175.00 |  |
| Who saw the 1^st^ symptom of concern | Family member | 48.0 | 204.0 | 0.17 | 26.50 | 213.00 | 0.34 |
|  | Health worker | 108.0 | 0.0 |  | 72.00 | 0.00 |  |
|  | Teacher/friends/neighbors | 49.0 | 49.0 |  | 16.00 | 25.00 |  |
| Belief of cause of 1^st^ symptom | Biomedical | 48 | 57 | 0.08 | 24.5 | 57 | 0.12 |
|  | Spiritual/cultural | 62.5 | 204 |  | 36 | 212 |  |
|  | Environmental/social | 43 | 48 |  | 21 | 54 |  |
|  | Don’t know | 68 | 56 |  | 26 | 44 |  |
| Have you heard of ASD before diagnosis | No | 60.0 | 201.0 | ***0.04*** | 36.00 | 213.00 | ***0.02*** |
|  | Yes | 40.0 | 72.0 |  | 23.00 | 68.00 |  |
| What do you believe is the cause of ASD | Don’t know | 49.5 | 173.0 | ***0.01*** | 28.50 | 172.00 | ***0.01*** |
|  | Biomedical | 45.0 | 72.0 |  | 24.00 | 57.00 |  |
|  | Supernatural | 72.0 | 180.0 |  | 53.00 | 192.00 |  |
| Knowledge of developmental milestones  Has stigma played a role in how you've determined to seek care | No | 61.5 | 74.0 | 0.96 | 36.00 | 60.00 | 0.90 |
|  | Yes | 48.0 | 204.0 |  | 24.50 | 213.00 |  |
|  | No | 42.0 | 60.0 | 0.06 | 19.00 | 57.00 | 0.06 |

**Supplementary Table 4: Child clinical characteristics and comorbidities associated with age and delay in diagnosis**

|  |  | Age at diagnosis  (Months) | | |  | Diagnostic delay  (Months) | | |
| --- | --- | --- | --- | --- | --- | --- | --- | --- |
|  |  | N=70 | Median | Range | P value | Median | Range | P value |
| Gender of child | Male | 60 | 50 | 204 | 0.13 | 30 | 213 | 0.26 |
|  | Female | 10 | 40.5 | 42 |  | 21 | 53 |  |
| Makes little or no eye contact | No | 9 | 48 | 62 | 0.92 | 24 | 52 | 0.82 |
|  | Yes | 61 | 49 | 204 |  | 30 | 213 |  |
| Fail to respond to their name being called | No | 19 | 54 | 66 | 0.70 | 36 | 68 | 0.85 |
|  | Yes | 51 | 48 | 204 |  | 26 | 213 |  |
| Rarely shares enjoyment for objects/activities with others | No | 9 | 62 | 46 | 0.78 | 34 | 48 | 0.64 |
|  | Yes | 61 | 48 | 204 |  | 25 | 213 |  |
| Has trouble understanding another’s feelings | No | 35 | 60 | 180 | ***0.03*** | 34 | 172 | 0.10 |
|  | Yes | 35 | 44 | 201 |  | 24 | 213 |  |
| Uses pronouns inappropriately | No | 63 | 48 | 204 | 0.16 | 25 | 213 | 0.46 |
|  | Yes | 7 | 72 | 61 |  | 36 | 68 |  |
| Echolalia (repeating the same words) | No | 38 | 45 | 204 | **0.02** | 24 | 213 | ***0.03*** |
|  | Yes | 32 | 60 | 174 |  | 36 | 174 |  |
| Has trouble maintaining social relationships with peers | No | 0 | . | . |  | . | . | . |
|  | yes | 70 | 48.5 | 204 |  | 26.5 | 213 |  |
| Shows absence of social gestures | No | 40 | 60 | 177 | *0.05* | 36 | 175 | 0.22 |
|  | Yes | 30 | 47 | 204 |  | 24.5 | 210 |  |
| Avoids physical contact | No | 46 | 50 | 204 | 0.60 | 30 | 210 | 0.90 |
|  | Yes | 24 | 48 | 177 |  | 24 | 175 |  |
| Has obsessive interests | No | 9 | 36 | 204 | 0.26 | 23 | 210 | 0.56 |
|  | Yes | 61 | 50 | 177 |  | 27 | 175 |  |
| Abnormal response to pain and/or sound | No | 4 | 32 | 38 | 0.05 | 11 | 32 | 0.11 |
|  | Yes | 66 | 49.5 | 201 |  | 28.5 | 213 |  |
| Forms rows regularly | No | 9 | 40 | 66 | 0.20 | 24 | 54 | 0.63 |
|  | Yes | 61 | 50 | 204 |  | 30 | 213 |  |
| Has abnormal movements | No | 5 | 50 | 60 | 0.80 | 37 | 54 | 0.55 |
|  | Yes | 65 | 48 | 201 |  | 25 | 213 |  |
| Language delay | No | 2 | 63 | 66 | 0.99 | 27 | 42 | 0.75 |
|  | Yes | 68 | 48.5 | 204 |  | 26.5 | 213 |  |
| Delay in walking | No | 48 | 48 | 72 | 0.07 | 24 | 69 | ***0.01*** |
|  | Yes | 22 | 60 | 197 |  | 36.5 | 207 |  |
| Regression of milestones | No | 39 | 48 | 204 | 0.58 | 24 | 212 | 0.30 |
|  | Yes | 31 | 54 | 177 |  | 36 | 175 |  |
| Selective of clothing | No | 48 | 45 | 84 | ***0.01*** | 24 | 69 | ***0.02*** |
|  | Yes | 22 | 60 | 194 |  | 37 | 212 |  |
| Comorbid ADHD | No | 38 | 43 | 204 | ***0.04*** | 23 | 212 | 0.05 |
|  | Yes | 32 | 56 | 81 |  | 36 | 69 |  |
| Comorbid convulsive disorder | No | 59 | 48 | 204 | 0.34 | 25 | 213 | 0.32 |
|  | Yes | 11 | 60 | 60 |  | 36 | 48 |  |
| Comorbid sleep problems | No | 64 | 48.5 | 204 | 0.83 | 26.5 | 213 | 0.57 |
|  | Yes | 6 | 54 | 51 |  | 31.5 | 51 |  |
| Enuresis/Encopresis | No | 58 | 49.5 | 180 | 0.85 | 28 | 175 | 0.63 |
|  | Yes | 12 | 48 | 195 |  | 26 | 207 |  |
| Comorbid Intellectual developmental disorder | No | 52 | 47 | 180 | ***0.02*** | 24 | 175 | ***0.01*** |
|  | Yes | 18 | 72 | 192 |  | 56 | 204 |  |
| Nutritional Challenges | No | 63 | 50 | 204 | 0.17 | 30 | 213 | 0.78 |
|  | Yes | 7 | 40 | 42 |  | 18 | 49 |  |
| Comorbid Learning disabilities | No | 67 | 48 | 204 | 0.07 | 26 | 213 | 0.45 |
|  | Yes | 3 | 96 | 42 |  | 48 | 30 |  |

**Supplementary Table 5: Age at ASD Diagnosis and Diagnostic Delay by Symptom Presentation**

|  |  | Age at diagnosis  (Months) | |  | Diagnostic delay  (Months) | | |
| --- | --- | --- | --- | --- | --- | --- | --- |
|  |  | Median | Range | P value | Median | Range | P value |
| First symptom of concern | Speech delay | 48.5 | 204 | 0.77 | 27 | 212 | 0.59 |
|  | Delayed/regression of milestones | 52 | 174 |  | 36.5 | 172 |  |
|  | Impaired sociocommunicative interaction-poor eye contact, difficulty making friends, etc. | 43 | 69 |  | 25 | 69 |  |
|  | Hyperactivity/obsessive interests | 40 | 63 |  | 23 | 39 |  |
| Symptom that prompted seeking help | Difficulty making friends/not responding to name | 37.5 | 36 | 0.07 | 13.5 | 41 | 0.06 |
|  | Aggression/self-harming behaviour/hyperactivity | 60 | 198 |  | 36 | 207 |  |
|  | Regression of milestones/developmental delays | 44 | 54 |  | 24 | 54 |  |
|  | speech impairment | 48 | 180 |  | 25 | 174 |  |

| **Column1** | **Column2** | **Column3** | **Column4** | **Column5** |
| --- | --- | --- | --- | --- |
|  |  | Type of Pathway | |  |
|  |  | Traditional/spiritual route | Healthcare based | |
|  |  | Count | Count | Chi square |
| First symptom of concern | Speech delay | 7 | 17 | 0.07 |
|  | delayed/regression of milestones | 6 | 12 |  |
|  | Impaired sociocommunicative interaction-poor eye contact difficulty making friends etc | 5 | 20 |  |
|  | Hyperactivity/obsessive interests | 1 | 2 |  |
| Symptom that prompted seeking help | Difficulty making friends/not responding to name | 1 | 3 | 0.06 |
|  | Agression/self harming behaviour/hyperactivity | 13 | 14 |  |
|  | regression of milestones/developmental delays | 2 | 13 |  |

**Supplementary Table 6: Association Between First Symptoms and Care-Seeking Pathways Among Children with ASD**

|  |  | Traditional/  spiritual route  (N=19) | Mainstream  Route  (N=51) | Chi square  P value |
| --- | --- | --- | --- | --- |
| First symptom of concern | Speech delay | 7 | 17 | 0.07 |
|  | Delayed/regression of milestones | 6 | 12 |  |
|  | Impaired sociocommunicative interaction-poor eye contact, difficulty making friends etc | 5 | 20 |  |
|  | Hyperactivity/obsessive interests | 1 | 2 |  |
| Symptom that prompted seeking help | Difficulty making friends/not responding to name | 1 | 3 | 0.06 |
|  | Aggression/self-harming behaviour/hyperactivity | 13 | 14 |  |
|  | Regression of milestones/developmental delays | 2 | 13 |  |
